# Determination of Neural Fiber Connections Based on Data Structure Algorithm

**DOI:** 10.1155/2010/251928

**Published:** 2009-12-22

**Authors:** Dilek Göksel Duru, Mehmed Özkan

**Affiliations:** Institute of Biomedical Engineering, Bogazici University, 34684 Istanbul, Turkey

## Abstract

The brain activity during perception or cognition is mostly examined by functional magnetic resonance imaging (fMRI). However, the cause of the detected activity relies on the anatomy. Diffusion tensor magnetic resonance imaging (DTMRI) as a noninvasive modality providing in vivo anatomical information allows determining neural fiber connections which leads to brain mapping. Still a complete map of fiber paths representing the human brain is missing in literature. One of the main drawbacks of reliable fiber mapping is the correct detection of the orientation of multiple fibers within a single imaging voxel. In this study a method based on linear data structures is proposed to define the fiber paths regarding their diffusivity. Another advantage of the proposed method is that the analysis is applied on entire brain diffusion tensor data. The implementation results are promising, so that the method will be developed as a rapid fiber tractography algorithm for the clinical use as future study.

## 1. Introduction

Functional magnetic resonance imaging (fMRI) serves to determine the brain activity during perception or cognition. BOLD contrast for fMRI is remarkable in cognitive neuroscience, surgical treatment planning, and preclinical studies in examining the main parameters such as the blood flow, blood volume, resting state connectivity, and anatomical connectivity within the brain [1]. To define the cause of the detected activity, the anatomy of the underlying tissue must be analyzed. The functional properties of the region of interests (ROIs) in the brain can be investigated by combination of different modalities such as diffusion tensor magnetic resonance imaging (DTMRI or DTI), ADC fMRI, and BOLD fMRI [2]. As a noninvasive imaging modality DTMRI helps identification and visualization of the fiber connections in the anatomy [3–5]. DTMRI is unique in its ability providing in-vivo anatomical information noninvasively. The potential of DTI is to make the determination of anatomical connectivity in the investigated brain regions by mapping the axonal pathways in white matter noninvasively [6].

The lack of a complete neural fiber map in literature makes the postprocessing of the data very important. Methods and updates are to be researched to define the fiber trajectories in the uncertainty regions where multiple fiber orientations cross within a single imaging voxel [7, 8]. Our proposed technique aims to track the white matter fibers according to data structure algorithm noniteratively and depending on the structural information of the underlying tissue. The proposed algorithm is based on two major processes. One is decision making and the other one is storing process. Decision making process is basically an operation based on comparison between the orientations of diffusivities of adjacent voxel pairs. In other words, it is the determination of the path to be traced for computing the neural pathways. The decision making involves setting a similarity measure having a constant scalar value for a subject. The voxels which succeeded to pass the threshold is stored in a data structure. This process is performed for all the adjacent voxel pairs in the examined brain MR images. So the study applies the method to the entire human brain DT images to construct maps of neural fibers in uncertainty regions.

## 2. Material and Methods

### 2.1. Principles of Diffusion Tensor Analysis

The Stejskal-Tanner imaging sequence is used to measure diffusion weighted images [3, 4, 9]. The diffusion tensor *D* is calculated from this raw data source at each point in the tissue formulated by the Stejskal-Tanner equation as [10, 11]

(1) $$S_{i}=S_{0}e^{-b{\hat{g}}_{i}^{T}D{\hat{g}}_{i}^{}},$$

where *S*_*i*_  is the  signal received with diffusion gradient pulses, *S*_0_ is the  RF signal received for a measurement without diffusion gradient pulses, *b* is the diffusion weighting factor, and |*g*| is the strength of the diffusion gradient pulses.

The diffusion tensor *D* is a real, symmetric second-order tensor, represented in matrix form as a real, symmetric 3 × 3 matrix [3, 4]. The six unique elements of the diffusion tensor *D* are calculated according to the three-dimensional Gaussian Stejskal-Tanner model as (2) by acquiring at least six diffusion-weighted measurements in noncollinear measurement directions *g* along with a nondiffusion-weighted measurement *S*_0_ [3, 4, 7, 12, 13]. On regular DTMR scans more than six diffusion-weighted measurements are taken which creates an over constrained system of equations solved using least square methods [9, 12, 14, 15]:

(2) $$\begin{array}{c} \left[\begin{array}{cccccc} x_{1}^{2} & y_{1}^{2} & z_{1}^{2} & 2x_{1}y_{1} & 2y_{1}z_{1} & 2x_{1}z_{1}\\ x_{2}^{2} & y_{2}^{2} & z_{2}^{2} & 2x_{1}y_{1} & 2y_{2}z_{2} & 2x_{2}z_{2}\\ \vdots & \vdots & \vdots & \vdots & \vdots & \vdots \\ x_{n}^{2} & y_{n}^{2} & z_{n}^{2} & 2x_{n}y_{n} & 2y_{n}z_{n} & 2x_{n}z_{n} \end{array}\right]\left[\begin{array}{c} D_{xx}\\ D_{yy}\\ D_{zz}\\ D_{xy}\\ D_{xz}\\ D_{yz} \end{array}\right]=\left[\begin{array}{c} -\frac{1}{b}\ln\;\frac{S_{1}}{S_{0}}\\ -\frac{1}{b}\ln\;\frac{S_{2}}{S_{0}}\\ \vdots \\ -\frac{1}{b}\ln\;\frac{S_{n}}{S_{0}} \end{array}\right]. \end{array}$$

Equation (2) equals a vector containing natural logarithmic scaled RF signal loss resulting from the Brownian motion of spins, and *x*_*i*_, *y*_*i*_, *z*_*i*_ denote the *n* gradient measurement directions. An orthogonal basis is the eigensystem of the symmetric matrix *D* by finding its eigenvalues and eigenvectors are calculated [16]. Principal component analysis (PCA) is used to perform the diffusion tensor analysis and compression. The diagonalization of the diffusion tensor as (3) results in a set of three eigenvalues *λ*_1_ > *λ*_2_ > *λ*_3_ representing the principal diffusion orientation in an investigated pixel [5, 8]. The eigensystem is defined by the eigenvectors *e*_*i*_ and the corresponding eigenvalues *λ*_*i*_ (4). The eigenvectors *e*_*i*_ represent the principal diffusion directions:

(3) $$\begin{array}{c} D_{x}{\vec{e}}_{i}=\lambda_{i}{\vec{e}}_{i}\;\left(i=1,2,3\right), \end{array}$$

(4) $$\begin{array}{c} \left|D_{x}-\lambda I\right|=0. \end{array}$$

Examining the raw data for every pixel, the eigensystem of *D* is calculated in each pixel. The eigensystem calculation for analyzed image data provides information about the diffusion distribution throughout the investigated image data. The first principal component *λ*_1_ shows the dominant diffusivity direction. The second and third principal components *λ*_2_ and *λ*_3_ provide information of the intermediate and the smallest principal diffusivity, respectively [17].

### 2.2. List Data Structure Implementation

**Figure 1:**
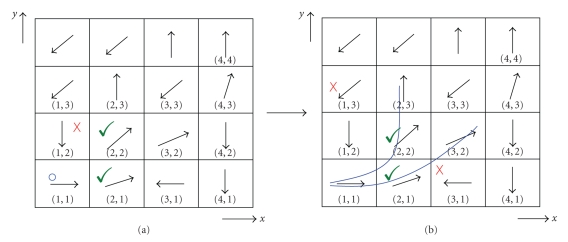
Sample synthetic eigenvector pattern. (a) (1, 1) is the starting node, where green checks represent the neighbors within the similarity measure.

The linear data structure used here helps to create a list of investigated region of interest eigenvectors where data item insertions and retrievals/deletions are made at one end, namely, the top of the list. A data item insertion is called pushing and removing is called popping the list. The created list can be called a linked list in which all insertions and deletions are performed at the list head (top) [18]. For each data item push, the previous top data item and all lower data items move farther down. When the time arrives to pop a data item from the list, the top data item is retrieved and deleted from the list. To clarify the implementation routine, application steps are explained on the synthetic data as in Figure 1. 

**Figure 2:**
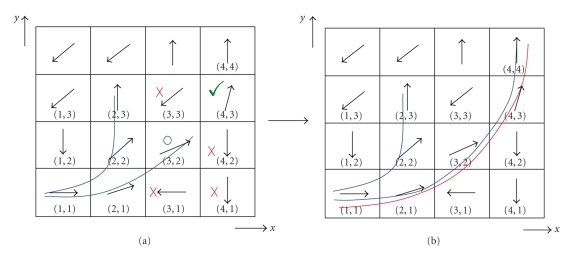
Listed data structure analysis results shown on sample pattern with its principal eigenvectors. Two possible resulting fiber paths are represented.

The starting point is selected as *x* = 1 and *y* = 1 as shown in Figure 1(a). This selected coordinate having the eigenvector [1, 0] is the bottom of the linked list. The predefined similarity measure is a set of angular thresholds *π*/*j*  (*j* = 4, 6, 12, 18, 20). Pixel (1, 2) is not within the limits of similarity measure *π*/4 (see Figure 1(a)). Pixel (2, 1) is stored in the stack on the top again in compliance with similarity. Top is now assigned to the new node. Next, pixel (2, 2) fulfilling the selected similarity measure is stored on the top of the list. The eigenvector [0.7*  *.07] with its neighboring pixels' eigenvectors is being compared for similarity. As a result, neighbors with coordinates (1, 3) and (3, 3) with both having the eigenvector [−0.7 −0.7] are eliminated (see Figure 1(b)). The implementation follows by pushing the coordinates (2, 3) and (3, 2) to the list. Pixel (3, 2) is popped. Then its neighbors are examined as in Figure 2(a). The routine follows by determining pixels matching with the predefined similarity rule *π*/4. The synthetic fiber path (represented in blue) is defined as a result as in Figure 2(b). 

Selecting the similarity measure as *π*/4 allows the pixel (2, 2) to be on the list as described above. But examining the pattern by a different try for a varying angular threshold such as *π*/6 or *π*/12, this pixel is not being assigned for the neighboring pixel list. As a result the track represented in red on Figure 2(b) is the outcome of the computational routine. The decision making here about to select a track follows regarding to the underlying tissue's structural information. 

The proposed approach relies on the assumption of the unique path description of an axon. Each element in the implementation represents a voxel in the ROI, and each voxel is related with its neighboring voxels. Regarding the neighboring voxel knowledge, the computation sorts the elements in the list for tracking, where the elements which do not fulfill the criteria are kept in a secondary matrix. While examining the investigated pattern pixelwise, the elements in the secondary matrix come up as potential neighboring pixels in question. The repeated check for if they are within the similarity criteria and if they belong to the fiber track gives the chance of a double check in the system. By that way, the neighboring is updated and a more secure resulting track is being defined and followed. The routine updates itself so that for the one selected starting node the first and second neighboring pixels are investigated and the computational routine is stretched to a wide range via this increased neighborhood.

## 3. Results

The proposed method is implemented on simulated fiber eigensystem to determine the predefined synthetic trajectories in Section 2.2. The output of the algorithm is in agreement with the visual inspection results as shown in Figure 2. Variation of the similarity measure causes major differences in the calculated neural path as seen in Figure 2(b). Small values of the similarity measure decreases the number of voxels in the solution which are defined by the decision making as neighboring voxels while increased similarity measure selections generate more well-defined and close results to the underlying tissue structure. 

**Figure 3:**
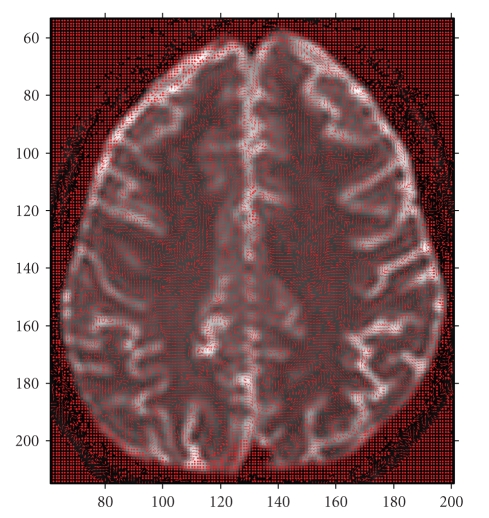
Calculated principal eigenvectors of the entire slice superimposed on axial brain MR image.

Following the promising results of the synthetic data implementations, the method is applied on real DT brain images. As explained in detail in Section 2.1 ((3) and (4)), the eigensystem of *D* is determined by PCA [19] and interpreted graphically as seen in Figure 3. 

**Figure 4:**
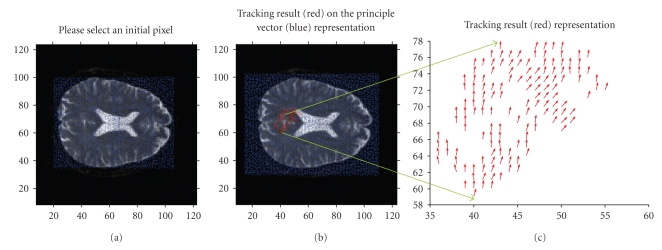
Fiber tracking results traced on axial slice with a similarity measure of *π*/20. (a) Starting point at [44, 70]. (b) Calculated neighboring pixels with related diffusivity mapped on entire eigenvector map. (c) Zoomed region of interest.

It is obvious that visual detection of any fiber path on the 2D axial MR image representing the eigenvectors is pretty hard unlike the simulated case. Therefore the developed linear list data structure algorithm is applied to the entire brain for neural fiber mapping. The search process of the pattern in the selected limits is completed in examining the eigenvectors of each pixel based on the predefined similarity measure. This examined data set sample might be a whole image data or a single ROI as in Figure 4. 

**Figure 5:**
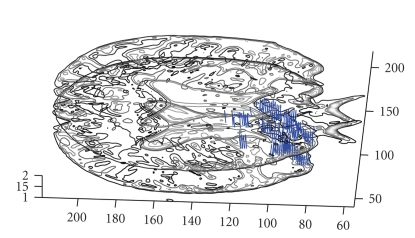
Tracking results of the implementation are represented on 2 consecutive slices.

**Figure 6:**
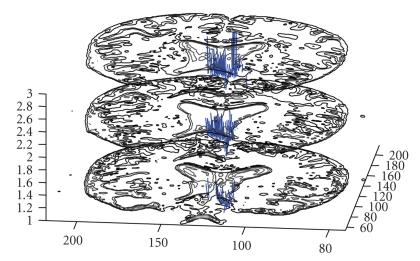
Fiber tracking results of the ROI close to inferior frontal lobe registered with the anatomic MR images.

The selection of the investigated brain region's size is directly related with the elapsed time of the computation. To be able to visualize the results of the algorithm, not the whole brain volume but only a selected and easily recognized region is computed. The results of such an example are represented in Figures 5 and 6 from different view angles in 3D. 

## 4. Discussion

Some modalities such as PET and fMRI makes it possible to map the brain functions noninvasively. A parallel fMRI experiment with DTI is promising for understanding the brain function in both neuroimaging and neuroanatomical techniques' sense [2]. The knowledge derived from the DTI make it possible to map the in vivo information of the human neural fiber pathways noninvasively. This is an important motivation in diffusion tensor analysis research. The postprocessing of DTI analyzing tools plays great role in determination of the anatomical structural maps of fiber tracts. To follow a fiber tract and to build a neural map, each voxel's trajectory is approximated by a set of computed lines in each voxel regarding their major diffusivity. Each resulting tract defines a curvature representing a small bundle of axons in the pathway. 

In the existence of fiber crossings and branches in an investigated ROI, the accuracy of the computed neural paths by DTI analyzing tools is unclear. One of the main limitations of diffusion tensor analysis relies on providing a solution for identification of the orientations of the brain fibers in uncertainty regions which is of great importance [3, 4, 8]. Therefore this problem arising in these so-called uncertainty regions is tried to be eliminated by different research groups [12, 20–23].

The aim of this study is to propose a rapid and reliable tracking algorithm which may eliminate the uncertainty region problem in DTI analysis. As seen in results, the synthetic fiber tracking implementation succeeded for predefined neural pathways. This motivated us to implement the algorithm on real diffusion tensor brain images. The computed tracts are found in agreement with the spatial visual inspection. Detailed anatomic information can be gathered via the computed tractography based on the Talairach atlas to become a gold standard, which is still missing. 

Future work relies on eliminating the tracking problems in the uncertainty areas by upgrading the proposed method so that the calculation will be implemented on neural system basis and physiological background. The results will provide the base to reliable brain mapping.

## 5. Conclusion

This work aims to develop a promising approach which may eliminate the uncertainties in DT-MRI fiber tractography reconstructions and enhance a neural mapping. The degree of uncertainty in fiber orientation is subject to change by the selection made for similarity measure to detect neighboring voxel pairs. The fiber tracking tools are limited to trajectory-based representations. Therefore the detection of the anatomical connectivity and reliable computation of the neural map should be applied carefully being aware of any mistaken result. 

It has been shown that linear list data structure gives promising analysis results in diffusion tensor fiber tract estimation. The identifying similarity measure varies in a range which is accepted in the means of anatomical fiber structure knowledge. Comparing the resulting tracts in synthetic eigenvector pattern with the known predefined pathways, the algorithm gives promising results and works well for the tracking purposes. The computed neural pathways varying with the change of the similarity measure cause to decrease or increase the number of the neighboring voxels for a selected starting voxel. The differing resulting pathways can be thought as an error of the method where it might be also in some cases the possible orientation of a fiber bundle in a wide range, which may be determined by an anatomical brain atlas, that is, Talairach atlas. 

Besides the existing algorithms the proposed technique provides the possibility to compute the whole eigensystem of the investigated brain volume. The neighboring voxel pair calculation compares the investigated node in every step of the algorithm within the entire image volume. Each voxel is checked for more than one trial in the total analysis. In that way the decision making of the algorithm becomes more precise.

## References

[1] Norris DG (2006). Principles of magnetic resonance assessment of brain function. *Journal of Magnetic Resonance Imaging*.

[2] Huettel SA, Song AW, McCarthy G (2004). *Functional Magnetic Resonance Imaging*.

[3] Basser PJ, Mattiello J, LeBihan D (1994). MR diffusion tensor spectroscopy and imaging. *Biophysical Journal*.

[4] Basser PJ, Mattiello J, LeBihan D (1994). Estimation of the effective self-diffusion tensor from the NMR spin echo. *Journal of Magnetic Resonance*.

[5] Basser PJ, Pajevic S, Pierpaoli C, Duda J, Aldroubi A (2000). In vivo fiber tractography using DT-MRI data. *Magnetic Resonance in Medicine*.

[6] Mori S, Crain BJ, Chacko VP, Van Zijl PCM (1999). Three-dimensional tracking of axonal projections in the brain by magnetic resonance imaging. *Annals of Neurology*.

[7] Basser PJ, Jones DK (2002). Diffusion-tensor MRI: theory, experimental design and data analysis—a technical review. *NMR in Biomedicine*.

[8] Le Bihan D, Poupon C, Amadon A, Lethimonnier F (2006). Artifacts and pitfalls in diffusion MRI. *Journal of Magnetic Resonance Imaging*.

[9] Basser PJ (1995). Inferring microstructural features and the physiological state of tissues from diffusion-weighted images. *NMR in Biomedicine*.

[10] Stejskal EO (1965). Use of spin echoes in a pulsed magnetic-field gradient to study anisotropic, restricted diffusion and flow. *The Journal of Chemical Physics*.

[11] Stejskal EO, Tanner JE (1965). Spin diffusion measurements: spin echoes in the presence of a time-dependent field gradient. *The Journal of Chemical Physics*.

[12] Westin CF, Maier SE, Mamata H, Nabavi A, Jolesz FA, Kikinis R (2002). Processing and visualization for diffusion tensor MRI. *Medical Image Analysis*.

[13] Kingsley PB (2006). Introduction to diffusion tensor imaging mathematics—part I. Tensors, rotations, and eigenvectors. *Concepts in Magnetic Resonance*.

[14] Pajevic S, Pierpaoli C (1999). Color schemes to represent the orientation of anisotropic tissues from diffusion tensor data: application to white matter fiber tract mapping in the human brain. *Magnetic Resonance in Medicine*.

[15] Sotak CH (2002). The role of diffusion tensor imaging in the evaluation of ischemic brain injury—a review. *NMR in Biomedicine*.

[16] Borisenko AI, Tarapov IE (1979). *Vector and Tensor Analysis with Applications*.

[17] Pierpaoli C, Barnett A, Pajevic S (2001). Water diffusion changes in wallerian degeneration and their dependence on white matter architecture. *NeuroImage*.

[18] Wirth N (1986). *Algorithms and Data Structures*.

[19] Goksel D, Ozkan M (2006). Towards rapid analysis of diffusion tensor MR imaging. *ESR Supplements*.

[20] Pajevic S, Basser PJ (2003). Parametric and non-parametric statistical analysis of DT-MRI data. *Journal of Magnetic Resonance*.

[21] Jones DK (2003). Determining and visualizing uncertainty in estimates of fiber orientation from diffusion tensor MRI. *Magnetic Resonance in Medicine*.

[22] Jones DK, Pierpaoli C Towards a marriage of deterministic and probabilistic tractography methods: bootstrap analysis of fiber trajectories in the human brain.

[23] Jones DK, Travis AR, Eden G, Pierpaoli C, Basser PJ (2005). PASTA: pointwise assessment of streamline tractography attributes. *Magnetic Resonance in Medicine*.

